# Do mtDNA Mutations Participate in the Pathogenesis of Sporadic Parkinson’s Disease?

**DOI:** 10.2174/138920209789503879

**Published:** 2009-12

**Authors:** E. Kirches

**Affiliations:** Department of Neuropathology, Otto-von-Guericke University, Leipziger Str. 44, 39120 Magdeburg, Germany

## Abstract

The pathogenesis of sporadic Parkinson’s disease (PD) remains enigmatic. Mitochondrial complex-I defects are known to occur in the substantia nigra (SN) of PD patients and are also debated in some extracerebral tissues. Early sequencing efforts of the mitochondrial DNA (mtDNA) did not reveal specific mutations, but a long lasting discussion was devoted to the issue of randomly distributed low level point mutations, caused by oxidative stress. However, a potential functional impact remained a matter of speculation, since heteroplasmy (mutational load) at any base position analyzed, remained far below the relevant functional threshold. A clearly age-dependent increase of the ‘common mtDNA deletion’ had been demonstrated in most brain regions by several authors since 1992. However, heteroplasmy did hardly exceed 1% of total mtDNA. It became necessary to exploit PCR techniques, which were able to detect any deletion in a few microdissected dopaminergic neurons of the SN. In 2006, two groups published biochemically relevant loads of somatic mtDNA deletions in these neurons. They seem to accumulate to relevant levels in the SN dopaminergic neurons of aged individuals in general, but faster in those developing PD. It is reasonable to assume that this accumulation causes mitochondrial dysfunction of the SN, although it cannot be taken as a final proof for an early pathogenetic role of this dysfunction. Recent studies demonstrate a distribution of deletion breakpoints, which does not differ between PD, aging and classical mitochondrial disorders, suggesting a common, but yet unknown mechanism.

## 1. INTRODUCTION

Parkinson’s disease (PD), originally described by the physician James Parkinson in 1817, is the second most common age-related neurodegenerative disorder after Alzheinmer’s disease. The central pathological feature is a profound loss of melanin-rich dopaminergic neurons in the substantia nigra (SN) pars compacta, which may exceed one half of the total neuronal population even at the beginning of symptoms. Since these neurons form the nigrostriatal pathway, which delivers dopaminergic input to the striatum, the severe loss of the neurotransmitter dopamine explains the central neurological symptoms of PD. Any disease causing striatal dopamine deficiency or striatal damage may elicit parkinsonism, characterized by tremor at rest, slow volontary movements, rigidity and poor balance, PD being the most common cause of parkinsonism (around 80% of cases). About 95% of PD cases are sporadic and the primary cause of neuronal death in the SN is not known. Only the small remainder is inherited and some monogenic forms can be explained by mutations of a handful of genes involved in protein folding, proteasome function, mitochondrial function and oxidative stress (reviewed in [1]). Although some of the encoded proteins are found in mitochondria or are associated with mitochondrial functions, it seems premature to declare any unifying hypothesis for common mitochondrial pathways in these genetic diseases. This will not be the subject of the present review.

Uncertainty of early pathogenetic events in the vast majority of sporadic PD cases inhibits the development of targeted therapies. Although L-dopa, a dopamin precursor applied for well established dopamine substitution therapy, can help most PD patients, its long term usage often causes the appearance of uncontrolled movements (dyskinesias), which severely affect the quality of life. It would thus be an intriguing challenge to uncover the early events of dopaminergic cell death at the molecular level. Although the discussion of PD is mainly focused on the central feature of dopaminergic cell loss in the SN, it should not be forgotten, that the pathology exceeds the nigrostriatal axis by far (for review see [2]). Neurodegeneration and Lewy bodies, characteristic proteineous inclusion bodies consisting of misfolded α-synuclein and various other misfolded proteins, are not restricted to the SN. They are also observable in noradrenergic, serotonergic and cholinergic systems, and occur in some areas of the cerebral cortex. Hippocampal neurodegeneration may contribute to late dementia in PD patients. This broader pathology should be kept in mind, even when weighing the latest exciting results obtained from the analysis of single dopaminergic SN neurons in human brains.

## 2. DYSFUNCTION OF THE ELECTRON TRANSPORT CHAIN IN PARKINSON’S DESEASE

Besides a basic role of protein misfolding, altered proteasomal degradation and cellular deposition of these proteins in the form of inclusion bodies (Lewy bodies), two other main hypotheses of nigral neurodegeneration focus on mitochondrial dysfunction and oxidative stress [1, 3, 4]. Since the mitochondrial electron transport chain (ETC) complexes I and III are main sources of reactive oxygen species (ROS) and mitochondrial enzymes are sensitive targets of ROS attacks, these latter two hypotheses are tightly linked. One route, by which ETC dysfunction, and mainly complex I inhibition, early became implicated in the pathogenesis of sporadic PD, was the observation that the intake of MPTP (1-methyl-4-phenyl-1,2,3,6-tetrahydropyridine) contaminated preparations of the street drug meperidine can cause a parkinsonian syndrome in humans, which is quite similar to PD [5]. A similar parkinsonism could also be elicited by MPTP in non-human primates, although the African green monkey was the only one, in which resting tremor was observed. A product of MPTP metabolism in dopaminergic neurons (MPP+) directly inhibits complex I predominantly in these cells [6]. Meanwhile, MPTP and the well-known complex I inhibitor rotenone, were exploited to establish rather artificial, but widely accepted toxic PD models in rodents [7-9]. Complex I inhibitors can even induce deposition of Lewy bodies [10] and thus link them as downstream events to a mitochondrial pathogenesis.

Shortly after the discovery of the parkinsonism inducing properties of MPTP, several reports in the late 1980’s and early 1990’s detected a moderately decreased activity of complex I and sometimes of other ETC complexes in the SN and in extracerebral tissues of human patients suffering from sporadic PD. Schapira and colleagues [11] analyzed homogenates of autoptic SN material from nine PD patients and nine controls matched with respect to age and post mortem delay. They found rotenone sensitive NADH-CoQ-oxidoreductase activity (complex I) to be decreased roughly by one third in the PD group and confirmed the result by a similar decrease in the ratio of rotenone sensitive NADH-cytochrome c-reductase versus succinate-cytochrome c-reductase. Normalization of complex I activity to the mitochondrial matrix enzyme citrate synthase (CS), which is meanwhile routinely used in studies of ETC function, would not have changed the results, since CS activity was nearly identical in PD and control groups. Succinate-cytochrome c-reductase activity did not differ between the groups, indicating no complex II defect in PD. In addition to these results, other authors observed a selective decline of some complex I polypeptides, but not of subunits of complexes III and IV, by immunoblotting [12]. Moderately decreased activities of complex I and other ETC enzymes in extracerebral tissues, such as skeletal muscle biopsies [13, 14] and platelets [15, 16] were also described in the early studies, suggesting a more widespread and generalized ETC dysfunction in sporadic PD, which may just elicit the strongest response in SN dopaminergic neurons due to their specific vulnerability. The technical problems, such as post mortem delay in SN studies, and the surprising extracerebral results initiated a controversial discussion [17]. The results obtained with muscle biopsies or blood platelets excluded at least the possibly confounding effects of post mortem delay. An ETC suppressing influence of L-Dopa treatment itself in the PD groups was excluded in studies analysing blood samples of PD patients, who were previously untreated with L-Dopa [16]. Inconsistent results obtained with respect to the presence or absence of ETC defects in extracerebral tissues and with respect the complexes involved, may have been caused by the use of classical enzyme measurements, which might not always detect moderate ETC dysfunction. More sophisticated techniques , such as inhibitor titrations, may be required [18] and were applied in later studies of skeletal muscle and cultured skin fibroblasts [19]. In the study of Wiedemann and colleagues [19], a moderate, but significant 30% reduction of NADH-cytochrome-reductase activity in skeletal muscle homogenates of PD patients was observed only after normalization to the significantly increased CS activity. CS increase itself may be interpreted in terms of compensative mitochondrial proliferation in skeletal muscle tissue, which is well known from classical mitochondrial disorders. In mitochondrial disorders, CS normalization of ETC enzyme activities is an accepted and established diagnostic procedure. No significant changes in CS normalized activities of ETC complexes II + III and IV were observed in PD pateints with direct enzyme measurements in muscle homogenates. Inhibitor titrations with amytal (complex I) and azide (complex IV) were then performed in saponin-permeabilized muscle fiber bundles, in order to determine the gradual inhibition of maximal respiration by increasing inhibitor oncentrations and to calculate flux control coefficients for the two complexes [18], i.e. their impact on electron flow through the ETC. This technique not only confirmed a complex I dysfunction, but detected a combined dysfunction of complexes I and IV in the majority of samples. Significantly increased flux control coefficients were also observed for both complexes in digitonin-permeabilized skin fibroblasts of PD patients, indicating ETC dysfunction in these cells. Mitochondrial defects could be restored in half of the analyzed skin fibroblast samples by 5 µM coenzyme Q [20]. The beneficial effects of coenzyme Q (CoQ) underline at least a participation of mitochondrial dysfunction at some stage of PD pathogenesis.

A further line of evidence implicating mitochondria and actually the mitochondrial genome (mtDNA) in PD pathogenesis, comes from ‘cybrid’ studies. While most polypeptides of all ETC complexes are transcribed from chromosomal genes, 13 of them are encoded in the small circular 16.6 kb mtDNA molecule, located in several copies within the mitochondrial matrix. This genome contains seven subunits of complex I and at least one subunit of all other ETC enzymes (with the exception of complex II) and of the mitochondrial ATP synthetase. Mitochondrial genes exhibit a much higher mutation rate as compared to nuclear genes and mtDNA is not protected by histones and exposed directly to ROS species generated during respiration. It was thus speculated that oxidative damage to mitochondrial DNA and resulting mutations may play a role for mitochondrial dysfunction in PD. So called ‘cybrid cells’ (*cytoplasmic hybrids*) were generated to uncouple potential effects of a damaged mtDNA from confounding effects of the nuclear background. In these cells, mtDNA of a tumor cell line (recipient) has been replaced by that one of a donor cell, derived from either a PD patient or a control. This goal is achieved by culturing the recipient cellline in subtoxic concentrations of an agent, which depletes the cells of their endogenous mtDNA, such as ethidium bromide or rhodamine-6-G. These so called rho-zero cells are then fused by polyethylene glycol treatment to either blood platelets of the donor (no nucleus) or to enucleated fibroblasts. In this way series of cell clones can be generated with identical nuclear background, but differing mtDNA. Using this technique, Swerdlow and colleagues demonstrated moderately decreased complex I activity (-20%), enhanced ROS production and enhanced MPTP sensitivity of neuroblastoma-derived cybrids, containing mtDNA from PD platelets [21]. Since only mtDNA was derived from the donor platelets in these experiments, they were interpreted as a hint for mtDNA transmission of the mitochondrial defect. These results were confirmed by the group of Schapira. These authors detected in addition a combined dysfunction of the two analyzed complexes I and IV in some of the cybrid clones [22]. In agreement with a disturbed mitochondrial function, lower ATP content and depolarized mitochondria were observed in teratoma-derived PD cybrids, together with higher LDH (lactate dehydrogenase) release and caspase-3 activity under unstimulated conditions, indicating enhanced cell death [23]. Even α-synuclein oligomerization, as a potential step towards Lewy body formation, could be defined as an mtDNA-transmissable downstream effect of mitochondrial dysfunction in PD cybrids. A more detailed discussion of the cybrid model can be found in the review of Trimmer and Benett [24].

Despite these hints for mtDNA transmission of mitochondrial dysfunction, direct sequencing of mtDNA from PD patients revealed no homoplasmic or heteroplasmic mutations, which were associated with the disease. This circumstamce fed the speculation that randomly distributed, somatic mutations may occur at low heteroplasmy levels (mutational loads), undetectable by direct sequencing. The mutations were thought to be caused by ROS (reactive oxygen species) generated in the ETC, a process which may be enhanced in dopaminergic neurons of the SN due to additional ROS sources in their metabolism. 

## 3. OXIDATIVE STRESS AND OXIDATIVE DNA DAMAGE

While oxidative stress is commonly accepted to occur in the SN of PD patients, its pathogenetic impact remains uncertain. It may just accompany a late stage of cellular degeneration as an unspecific feature of dying neurons, or it may reflect or actually elicit mitochondrial dysfunction at a relatively early time, when the decision of an individual cell’s fate is being made. In the latter scenario, it would be worth to test pharmacological interventions, capable of restoring ETC function and scavenging ROS. A theoretically promising molecule for this purpose is the flexible electron carrier and antioxidant ubiquinone (coenzyme Q_10_, CoQ_10_). While it indeed protects primary dopaminergic neurons *in vitro* against MPTP induced cell death [25] and seems to partially restore complex I function in tissues of PD patients [26], the results of clinical trials, assessing neuroprotection, have been contradictory. Some small-sized studies found beneficial effects in UPDRS scores (Unified Parkinson Disease Rating Scale) or visual symptoms [27-29], but benefitial effects were not generally confirmed in all studies [30]. Although neuroprotective efficacy of antioxidative treatments in humans may be questionable, the fact of oxidative changes in lipids, proteins and nucleic acids in the SN of PD patients has been confirmed by many investigators (for review see [4]). 

**Fig. (1):**
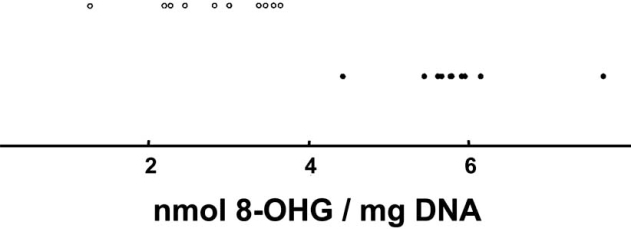
Among various DNA adducts quantified in the study of Alam and colleagues [34], selectively 8-OHG exhibited a pronounced and significant increase in the SN of 10 PD patients (filled circles) as compared to the SN of 10 aged-matched controls (open circles). The distributions of the determined concentrations (normalized to mg DNA) did not even overlap between the groups. Modified according to [34].

Malonaldehyde, a marker of lipid peroxidation, was found to be increased, while the total amount of polyunsaturated fatty acids was decreased [31]. The compound 4-hydroxy-2-nonenal, which is interpreted as a product of the peroxidation of membrane-bound arachidonic acid in the SN of PD patients, was shown to built protein adducts [32], which also occur in α-synuclein and alter its aggregation properties [33]. This peroxidation product may thus support Lewy body formation, a morphological hallmark of PD. Carbonyl modifications of soluble proteins are also increased in PD [34, 35], as well as nitration and nitrosylation of proteins, including α-synuclein [36]. Protein carbonyls of complex I subunits were enhanced in brain mitochondria from PD patients [37], indicating oxidative damage of this complex itself, which may partially explain the reduced activity. On the level of DNA, the marker 8-hydroxy(deoxy) guanosine (8-OHdG/8-OHG) demonstrates the enhanced oxidative damage in the SN of PD brains [34, 38]. It is of interest, that the increase of this oxidation product in the SN of human PD patients was rather selective (Fig. 1), while no significant changes were observed in other oxidation– or deamination products [34]. The base modification 8-OHG is not a result of direct reactions of DNA with superoxide or hydrogen peroxide [39-41], which are liberated as the primary ROS species during electron transport and superoxide dismutase (SOD2) reaction, respectively. Hydroxyl radicals, generated in the presence of ferrous iron by the Fenton reaction, can hydroxylate DNA bases, but would have been expected to generate a broader spectrum of oxidation products in PD. Since this broader increase was not observed, the precise mechanism of enhanced oxidative DNA damage remained uncertain.

Oxidative stress markers have been meanwhile described by several studies even in extracerebral tissues and bodily fluids of PD patients [38, 42-45], including an increase of 8-OHdG/ 8-OHG in the cerebrospinal fluid [46, 47]. This points towards a more generalized oxidative stress affecting and probably mutating nuclear and mitochondrial DNA. 

Besides the possible pathogenetic impact of oxidative stress in the dopaminergic neurons, a second important question cannot be answered sufficiently at present: the origin(s) of oxidative stress. Is it mainly caused by enhanced ROS production of inhibited ETC complex I or by enhanced ROS generation from other metabolic sources, such as dopamine metabolism? Or may it be caused by a lowered antioxidative defense, allowing higher ROS concentrations even under conditions of unaltered production? Is the primary pathogenic role of complex I in the dopaminergic neurons that one of an important ROS source or of an important ROS target? What comes first: ETC damage or oxidative stress? 

One early biochemical alteration observed in the SN of PD patients, a loss of reduced glutathione (GSH), may support an important role of weakened antioxidative defense [48, 49]. In the dopaminergic celllines PC12 and N27, complex I inhibition was provoked as a secondary event following experimental GSH depletion [50, 51]. This suggests that complex I dysfunction in the SN of PD brains may rely on or at least be enhanced by GSH depletion. On the other hand, it has been suggested that chronic mild complex I inhibition may reduce the concentration of the antioxidant GSH by enhanced ROS production. The first ROS species generated by complexes I and III, superoxide, is metabolized to hydrogen peroxide by manganese-dependent superoxide dismutase (SOD2) in the mitochondrial matrix. Hydrogen peroxide is partially detoxified by glutathione peroxidase in a GSH consuming reaction. Disturbed ETC function and oxidative stress may be tightly linked and enhance each other.

If ETC dysfunction and oxidative stress occur also at extracerebral sites in PD, as suggested by several reports, why do mainly dopaminergic neurons die? One important factor to explain selective vulnerability of the dopaminergic cell population, is the relatively high baseline of ROS production due to dopamine (DA) metabolism. Both, enzymatic DA degradation by monoamine oxidase and intracellular autooxidation of DA yield H_2_O_2_ [52, 53], while the latter process in addition generates superoxide and a reactive DA-quinone [54, 55]. The DA-quinone in turn may directly participate in nucleophilic addition reactions with sulfhydryl groups, thus damaging neuronal proteins and supporting GSH depletion [56, 57]. Reactive quinones and semiquinones can be built in addition during cyclooxygenase-2 catalyzed reactions of DA [58]. 

Since ROS generation in the ETC may induce mtDNA mutations, which in turn may inhibit ETC complexes and enhance mitochondrial ROS production, a long debate dealt with a possible ‘vicious cycle’, which may lead to a self-accelerating feedback between oxidative DNA damage and mutagenesis, leading to an accelerated (non-linear) accumulation of mutated mtDNA in aging dopaminergic neurons. Since accumulation of such mutations, widely distributed over the mitochondrial genome, offered an attractive explanation for the age-dependent onset and progressive worsening of PD and some other neurodegenerative diseases, mtDNA came into the focus of research. 

## 4. HETEROPLASMIC POINT MUTATIONS OF LOW ABUNDANCE

In the 1990s some sequenced mtDNA mutations, i.e. inherited homoplasmic or heteroplasmic mutations representing a relatively high percentage of total mtDNA, had been suspected to be more frequent in Caucasian PD patients as compared to haplogroup-matched controls (belonging to the same branch of the mtDNA evolutionary tree). However, studies with larger numbers of well characterized or pathologically proven PD cases could not confirm any of these predicted PD associated mtDNA mutations [59, 60], rejecting the idea of a maternally inherited mtDNA contribution to complex I dysfunction. Even more sophisticated approaches, designed to detect heteroplasmic mtDNA contributions between 5 and 10% in two complex I genes, did not yield any significant differences between PD and control groups in DNA isolated from SN or platelets [61].

However, these results did not exclude a higher burden of widely distributed heteroplasmic mutations in PD tissues, which may reach only extremely low heteroplamy levels at any given base position and thus require highly sensitive detection techniques. Such mutations of low abundance would actually be expected, if somatic mutagenesis was driven by oxidative stress. PCR-cloning of mtDNA with a proofreading polymerase and subsequent sequencing of clones is a technique, which allows to further enhance the sensitivity of mutation detection. It is mainly limited by the endogenous error rate of the chosen proofreading polymerase and by the fussiness of this rather time-consuming method. Due to the latter circumstance, the method usually allows only small sample sizes, and results must be interpreted with caution. A few studies used this technique to analyze various tissues of PD patients and age-matched controls. In their first study, Smigrodski and colleagues compared all seven complex I genes of the mtDNA in six PD patients versus six age-matched controls [62]. The authors used autoptic frontal cortex tissue, since they had previously found frontal cortex to manifest the complex I defect and suggested a strong dilution of mutated DNA in tissue homogenates of the SN, due to the severe loss of dopaminergic neurons in the end stage disease, represented in autoptic brain tissue. Indeed, this argument applies to the early biochemical studies too (see above), which detected only moderate complex I dysfunction in the SN. By sequencing a mean of 94.4 clones of every complex I region per case, the authors were able to achieve a sensitivity of roughly 1% heteroplasmic DNA. Although they did not detect a significant difference in the total mutation burden between the two groups, amino acid exchanges in a few codons of five subunits were restricted to PD samples, suggesting disease-specifity. Due to the low number of tissue samples, a retrospective narrowing of the viewpoint like this, includes the risk of selecting artificial differences rather than identifying possible hot spots of somatic mutations in PD. Most of the selected codons exhibited a mutation frequency just above the detection limit, i.e. one mutation in all seqnenced clones. Nevertheless, the authors analyzed a small candidate region between codons 120 and 150 of the ND5 subunit in more detail in a second study [63]. This study included frontal lobe brain tissue from eight idiopathic PD patients and eight controls. Methods used were identical to the first study, but analysis was restricted to the ND5 gene. Fifteen out of 16 samples could be correctly assigned to the two groups on the basis of mutation data, since the mutations in the selected region (ND5, codon 124–148) were restricted to the PD group with the exception of one control. This result suggested that a selective target region for low abundance mtDNA mutations in idiopathic PD might have been identified. Again it should be emphasized that all but one of the observed mutations occurred at a heteroplasmy level just above the detection limit of the assay.

Stimulated by these results, our laboratory analyzed with a similar cloning technique skeletal muscle from five idiopathic PD cases of the akinetic-rigid type, which had suffered from PD between two and thirteen years, and had previously been shown to exhibit a complex I dysfunction in a skeletal muscle sample, a small frozen part of which was now used for DNA preparation [64]. The mean percentage of mutated clones was 2.1% in PD versus 2.7% in age-matched controls. The exclusion of silent mutations shiftet the percentages to 1.7% versus 1.9%, indicating no increase in PD. The results were roughly in accordance with the overall mutation frequencies observed in the frontal cortex tissue by Smigrodski and colleagues [62], but did not reproduce the ND5 region between codons 120 and 150 as a hotspot for low abundance mutations in complex I deficient PD tissue. The highest frequency of mutated clones (6.1%) was actually observed in a control sample without any known history of neurodegenerative or neuromuscular disorder. Although the difference between the studies may reflect the different tissue sources, it is unlikely that low abundance ND5 mutations generally play a major role in complex I defects outside the SN. 

Another study used the so called ‘double PCR and digestion (DPD)’ method to measure specific base exchanges in mildly ETC deficient skeletal muscle of nine PD cases and nineteen controls at two specific positions of the mtDNA (np 7445 and 8993). Both base substitutions are known to be pathogenic due to their proven role in classical mitochondrial disorders, but both sites are located outside of complex I genes, i.e. in subunits of cytochrome c oxidase and ATP-synthetase. The method was originally used to investigate mtDNA point mutations in aging muscles [65] and had been used to demonstrate mutations of low abundance in spinal cords of patients with another neurodegenerative disease, i.e. amyotrophic lateral sclerosis [66]. The method exploits a step of endonuclease digest to destroy most wild type PCR products, in order to enrich the low abundant mutations, prior to a second PCR step and quantification of the bands. The method revealed a slight, but significant increase of the two selected mutations in PD muscles [67]. Altogether, studies concerning low abundant point mutations analyzed in homogenates of various PD tissues, delivered conflicting results.

## 5. RELEVANT LOADS OF MITOCHONDRIAL DNA DELETIONS IN AGING DOPAMINERGIC NEURONS

### 5.1. Age-Dependent Increase of the Common Deletion in Brain Homogenates

Aging is the main risk factor for developing idiopathic PD. Another type of mtDNA mutation of low abundance in the human brain had been clearly associated since the year 1992 with the process of aging: the so called ‘5 kb common deletion’. Using dilution-PCR, Corral-Debrinski and co-workers demonstrated that this large scale deletion accumulates dramatically in the putamen of aged persons, but to a lesser extend also in occipital, frontal and temporal cerebral cortex, while the cerebellum was largely devoid of deleted mtDNA [68]. A similar heterogeneity was reported in the same volume of ‘Nature Genetics’ by Soong and colleagues, who observed a dramatic age-dependent accumulation of the deletion in SN, caudate nucleus and putamen and a much smaller increase in various other brain regions, including parietal and frontal cortex, globus pallidus, hippocampus, centrum semiovale and thalamus [69]. Again, the lowest levels were measured in the cerebellar grey matter. A few later studies largely confirmed this aged-dependend increase of the ‘common deletion’ in the human brain, including unpublished observations in our laboratory obtained by Real-Time-PCR. The deletion affects several genes of complexes I and IV and of the ATP-synthetase, as well as several tRNAs needed for translation of mitochondrial transcripts. If the mutational load reaches a functional threshold, it becomes clearly pathogenic as underlined by some classical mitochondrial disorders. In these disorders (chronic progressive external ophthalmoplegia, Kearns-Sayre-syndrome), the ‘5 kb common deletion’ and other large scale rearrangements can be usually found at high levels in skeletal muscle tissue. In contrast, the deletion burden in aging human brains seems to be usually below 1% of total mtDNA, although the quantitative estimations in such PCR-studies differed in detail. However, there is no doubt that even the highest reported heteroplasmy levels were far below the threshold expected to cause a significant decline of ETC enzyme activities, which was estimated to be around 60% [70]. A functional impact of deletions in age-dependent neurodegeneration may be considered, if various clonally expanding deletions accumulate in the same brain region, the measured ‘common deletion’ being just the tip of the iceberg. Alternatively and more likely, a true functional impact of deletions may simply be masked in tissue homogenates, if a high mutational burden is restricted to a specific cell population. Although the mechanism for the accumulation of deletions remains unclear, oxidative stress in metabolically active brain regions was suggested to play a role. It is interesting to stress the high content of dopaminergic neurons in the most severely affected regions: SN, caudate nucleus and putamen. Considering this situation, it was necessary to develop PCR-techniques to analyze the mitochondrial genome of single dopaminergic neurons, derived from PD patients or elderly individuals without neurodegenerative disease.

### 5.2. Functional Levels of Clonal Deletions in Microdissected Dopaminergic Neurons 

Fourteen years after the initial studies described above, a major breakthrough was achieved independently by two groups in the year 2006, who demonstrated the age-dependent accumulation of clonal somatic mtDNA deletions in individual nigral neurons, which reached relevant levels to cause ETC dysfunction and eventually neuronal death.

**Fig. (2):**
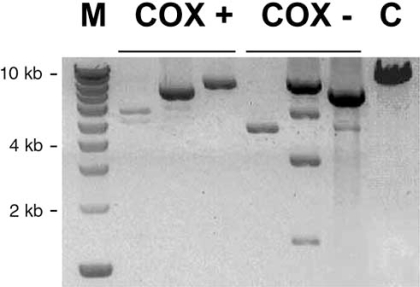
Gel showing results of single-cell PCR detection of various clonal large-scale deletions between 1 and more than 8 kb in size, which occurred in single COX-positive or COX-negative dopaminergic neurons of the SN of a 75-year old subject, modified according to [71]. M = 1 kb ladder, C = control muscle representing the fragment length generated from intact mtDNA.

Bender and colleagues [71] examined a cohort of individuals suffering from PD, who had a history of movement disorder and prominent dementia, as well as widespread Lewy body formation. Most important, some of these individuals exhibited a less pronounced neuronal loss in SN, a situation which is considered to be favourable for mtDNA studies. The authors first established a histochemical method for the detection of ETC deficient neurons, based on a double staining for ECT enzymes cytochrome c oxidase (COX) and succcinate dehydrogenase (SDH). This technique is well established to detect COX deficient muscle fibers in classical mitochondrial disorders. It is a good surrogate marker for cells with deletions or other severe mutations of the mtDNA, which affect multiple ETC complexes. Since no SDH subunit is encoded by the mitochondrial genome, SDH counterstain yields bright blue cells in case of COX deficiency, which is of special advantage in melanin containing brownish neurons. A significantly greater proportion of COX-negative neurons occurred in the SN of PD brains as compared to age-matched controls. Sequencing of the whole mitochondrial genome, amplified from nine individual COX-negative neurons of PD patients, revealed no relevant point mutations explaining COX deficiency, such as base exchanges in tRNAs or in conserved COX codons. The low impact of point mutations in individual neurons was later confirmed by a separate study of the same group [72]. Next, the authors pooled 50 laser-microdissected neurons from frozen SN. Long-range PCR, designed to detect all large-scale deletions irrespective of their localisation within the genome, detected only mtDNA with deletions (∆mtDNA) in PD patients and age-matched controls. This suggested a high percentage of ∆mtDNA copies, although long-range PCR is not quantitative and certainly favours shorter templates. At least microdissected glial cells revealed only full length products, supporting the reliability of the method. Applying the same technique to single microdissected neurons, multiple large-scale deletions of different length were found in individual cells (Fig. 2). This result must be interpreted in terms of a clonal expansion within the individual cells, most likely reflecting somatic mutations. Clonality was confirmed by sequencing of four deletion breakpoints.

To allow reliable quantification of ∆mtDNA, the authors took advantage of the fact that nearly all deletions of the mitochondrial genome occur in one half of the circular molecule. This allowed a relative PCR-quantification of two short fragments, representing either the deletion-prone major arc of mtDNA (complex I gene ND4) or the commonly unaffected half of the molecule (ND1). Twenty-five microdissected nigral neurons with normal COX activity from PD patients were compared with age-matched controls. High levels of the mutations were observed, which seemed to be somewhat higher in PD patients (mean value 52.3%) as compared to controls (43.3%), although the difference was not significant (p = 0.06). These mean levels were near to the suggested functional threshold around 60%, which was exceeded in some cells. Moreover, the mean level of deletions was significantly higher in COX-negative cells as compared with COX-positive neurons, underlining the functional impact. The formerly described age-dependency of deletion burden in the brain [68, 69] was confirmed in microdissected nigral neurons. The same PCR assay from hippocampal neurons did not reveal such relevant mutational burden.

**Fig. (3):**
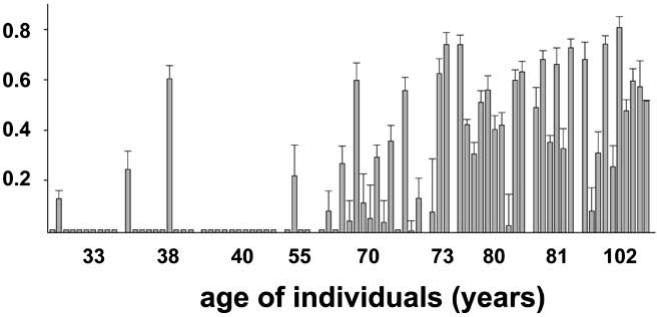
Proportion of ΔmtDNA in single microdissected dopaminergic neurons of the SN (represented by the individual coloums) plotted against the age of the subjects, which ranged from 33 to 102 years. Modified according to [73].

Largely the same results with respect to aging and functional COX decline were obtained in an independent study of Kraytsberg and colleagues, who analyzed brains of individuals between 33 years and 102 years of age with similar methods [73]. The study was published in the same volume of ‘Nature Genetics’. In many of the microdissected dopaminergic nigral neurons, the percentage of ∆mtDNA exceeded the suggested threshould of 60%. This was the case in a large fraction of neurons for individuals above their eigth decade of life (Fig. 3), a feature which corresponds nicely to age-dependent PD risk. In several other neuronal cell types, such as pyramidal cells of the cortex, cerebellar Purkinje cells and large neurons of the dentate nucleus, ∆mtDNA was usually undetectable. A detailed analysis in a single 80-year old individual with a high percentage of COX-negative nigral neurons revealed that these cells usually exhibited a mutational burden above 60%, while COX-positive neurons did not. Another recent analysis of microdissected neurons in aged individuals and Alzheimer patients revealed that the mutational load in the SN generally exceeded that one in frontal cortex, but also in dopaminergic neurons of the putamen [74]. Disease status played no role. 

Taken together, the reports clearly established a connection between progressive mitochondrial genome deletion during aging and developing ETC dysfunction in the SN, which may participate in neuronal cell loss in this brain region They deliver exiting hints, but do not demonstrate unequivocally that this process of deletion-driven mitochondrial dysfunction is accelerated in PD patients.

## 6. POSSIBLE ORIGINS OF CLONAL DELETIONS AND THEIR POSSIBLE ROLE IN PARKINSON’S DISEASE

The nature of mitochondrial DNA deletions in nigral neurons still remains unclear. A comparison of breakpoints was performed in laser-microdissected individual neurons of brains from PD patients, aged individuals and one patient suffering from autosomal progressive external ophthalmoplegia (PEO) due to mutations in the polymerase-γ (POLG) gene. Polymerase-γ copies the mitochondrial genome. Mutations of this gene in autosomal PEO cause a fragility of mtDNA, which leads to multiple ∆mtDNA-species e.g. in skeletal muscle of the patients. Moreover, the disease is sometimes associated with parkinsonism. In all groups, most deletions (mean value 79%) were flanked by perfect or imperfect short direct repeats, suggesting a common mechanism [75], irrespective of the disease scenario. Based on an early model of mammalian mtDNA replication [76], deletions had been originally discussed to occur during replication by slipped mispairing between direct repeats [77, 78]. Even in the light of novel modified models [79] the replication process remains a hypothetical source of large-scale deletions. Alternatively, they could be generated by recombination during repair of damaged DNA [80]. Since dopaminergic neurons of the SN are exposed to enhanced ROS levels and reactive quinones, oxidative stress is hypothesized to play a role. It may either impair replication or induce DNA repair mechanisms, which favour rearrangements. The latter hypothesis is supported by experiments showing that artificially generated double strand breaks (DSBs) induce large-scale deletions in mice [81, 82]. Double strand breaks can result from stalling of the replication fork, but also from oxidative stress, offering a hypothetical link to dopaminergic metabolism. 

The elegant mouse model, recently developed by the group of C. Moraes, allowed to generate DSBs in the mouse brains in a timely and spatially controlled manner and to observe accumulation of ∆mtDNA-species and functional defects over time [82]. The authors expressed a mitochondria-targeted gene for the restriction endonuclease PstI in the brains, which was controlled by a Tet-promoter. This approach simulated naturally occurring DSBs in the mitochondrial genome, if mice were grown in the absence of doxorubicin (Dox). In this case mice developed a neurodegenerative ‘limb-clasping’ phenotype, which was suppressed in mice grown with Dox within the first three weeks of life. Only continuously Dox treated mice reached a normal lifespan. As expected, expression of the restriction enzyme reduced the ratio of intact mitochondrial DNA versus nuclear DNA in striatum, hippocampus and cortex and induced a decline of a catalytic COX subunit and of COX activity. The authors detected many large-scale deletions of mtDNA, although smaller ones (longer PCR products) were expected to be missed by the technique. A closer characterization of these DSB-induced deletions revealed that 59% of them contained no direct repeats and were thus more likely to be generated by non-homologous end-joining than by homologous recombination involving repeats. Even after timely limited PstI expression, the brains continued to accumulate ∆mtDNA, suggesting clonal expansion. This elegant model supports the hypothesis that both, repeat-free and repeat-bearing deletions may be caused by unknown oxidative damage-induced repair mechanisms, while replication errors may contribute to the latter type.

Despite the exciting progress made possible by sophisticated single-cell PCR techniques and mouse models in the last three years, important questions arising from those studies cannot be answered at present. Currently, one of the most important of these unresolved questions is: what may drive a slightly faster accumulation of ∆mtDNA in a minority of individuals, who are then condemned to develop full-blown PD? May a short incidental increase in oxidative stress and DSBs (e.g. by environmental noxes) be sufficient to initiate a fatal process of ∆mtDNA accumulation, leading to ETC dysfunction and finally neuronal death? What makes the difference between PD and normal aging?
